# Genome-Scale Studies of Aging: Challenges and Opportunities

**DOI:** 10.2174/138920212803251454

**Published:** 2012-11

**Authors:** Mark A McCormick, Brian K Kennedy

**Affiliations:** 1 Buck Institute for Research on Aging, Novato, CA, USA; 2 Aging Research Institute, Guangdong Medical College, Dongguan 523808, Guangdong, P.R. China

**Keywords:** Aging, Genomic, High-throughput, Lifespan, Whole-genome, Yeast, *C. elegans.*

## Abstract

Whole-genome studies involving a phenotype of interest are increasingly prevalent, in part due to a dramatic increase in speed at which many high throughput technologies can be performed coupled to simultaneous decreases in cost. This type of genome-scale methodology has been applied to the phenotype of lifespan, as well as to whole-transcriptome changes during the aging process or in mutants affecting aging. The value of high throughput discovery-based science in this field is clearly evident, but will it yield a true systems-level understanding of the aging process? Here we review some of this work to date, focusing on recent findings and the unanswered puzzles to which they point. In this context, we also discuss recent technological advances and some of the likely future directions that they portend.

## INTRODUCTION

Aging, characterized by a finite organismal lifespan and a gradual decrease in function at the organismal and molecular level, is a fundamental unsolved problem of biology [1, 2]. Although at one time it may have been possible to imagine that aging represented only an entropic process similar to the malfunctioning of a piece of machinery, it has now become clear that aging and lifespan are genetically malleable, and are regulated in much the same way as many developmental processes. The social and economic costs of aging and age-related diseases cannot be overstated. There is an on-going dramatic global increase in the number of individuals over the age of 65, with most having one or more chronic disease conditions.

Several signalling pathways affecting lifespan have been discovered, which are in some cases conserved across multiple species ranging from yeast to humans [3-7]. Among the foremost are the TOR [8-11] and the Insulin/IGF signalling [12-14] pathways. Once it became clear that single-gene mutations and deletions could dramatically affect lifespan in well-studied model organisms [12, 13, 15], several genome-wide screens were undertaken in an attempt to identify as complete a set of longevity genes as possible. These screens have been done in the budding yeast *S. cerevisiae*, the nematode *C. elegans*, and fruit flies, all of which will be discussed below (see also the accompanying review in this issue by Bennet *et al*. [16]), as well as in *E. coli* [17]. Here we will restrict ourselves largely to a survey of recent work in non-human model organisms. The results of these studies have begun to shed light on many of the genes and processes that can affect lifespan and healthspan. At the same time, there are discordant results in some of the screens that should overlap. This and other data suggest that these screens are not saturated; that is, there are more lifespan and healthspan influencing single gene mutations and deletions to be found. Even so, it should be possible to continue combining large sets of existing data to make additional genome-scale inferences about processes involved in aging. In addition, new techniques to allow faster high-throughput lifespan assays may permit screening this phenotype at a much higher resolution across whole genomes in the near future. Simultaneously, rapid progress in bioinformatic approaches should make it possible to extract even more genome-scale inferences about lifespan-affecting loci by combining existing large data sets.

## GENOME-SCALE SCREENS FOR INCREASED LIFE SPAN

Aging in the relatively simple eukaryote budding yeast *Saccharomyces cerevisiae* has been extensively studied and is complemented by research in other fungi, including *Schizosaccharomyces pombe* [18, 19], *Podospora anserina* [20-22], and *Neurospora crassa* [23]. *S. cerevisiae* lifespan has been measured in at least two ways. Chronological lifespan (CLS) measures the continued viability of yeast in a post-replicative culture over time, and as such may be a reasonable model for the survival of long-lived non-dividing cells in metazoans [24-27]. Replicative lifespan (RLS) measures the number of daughter cells that a single yeast mother cell can give rise to by budding, and may be a better model for the senescence of continuously dividing cells such as stem cells [28, 29]. For chronological lifespan, a genome-wide screen of a portion of the *S. cerevisiae* deletion collection [30] has been completed [31]. This screen uncovered an overrepresentation of deletions affecting the TOR signalling pathway among those strains exhibiting increased CLS. The TOR pathway is activated by reduced intracellular amino acid levels [32], particularly glutamine levels [33], and this screen showed CLS extension arising from the deletion of several genes involved in glutamine metabolism, as well as from treatment with two known TOR inhibitors, rapamycin [34-36] and methionine sulfoximine [37]. A more recent microarray bar-code based whole genome screen for CLS phenotypes identified genes involved in purine biosynthesis and import, as well as several novel, previously unstudied genes, whose deletion extended yeast CLS [38]. Interestingly, the TOR pathway was also identified in a partial screen of the same collection for yeast gene deletions which extend *replicative* lifespan, along with *sch9Δ* [9]. This screen of ~4800 single gene deletion strains for increased RLS has now been completed (Kaeberlein and Kennedy labs, manuscript in preparation), giving a snapshot of this phenotype at near genome-scale (all viable deletions). Additional study has already been devoted to several sets of gene deletions discovered during the progress of this screen. This has revealed a very large overrepresentation for components of the ribosome [39, 40], which had been independently noted to extend both yeast RLS [41] and *C. elegans* lifespan [42]. It has also revealed a role for the proteasome in yeast RLS [43], and again the proteasome has been shown to affect both *C. elegans* lifespan [44-47] and *S. pombe *CLS [48, 49]. One future direction for this work should focus on the phenotype of lowered expression for those genes whose deletions are inviable, for example by the use of the tetracycline repressible promoter collection of essential yeast genes [50]. The fact that not all previously studied lifespan-extending deletions appeared in the RLS screen suggests that there is some room for further saturation, perhaps by using very recent advances in high throughput RLS screening that will be discussed below. Similarly, the incomplete overlap of findings from the two CLS screens, though easily ascribable to their very different methodologies, suggests that additional CLS affecting gene deletions likely remain to be identified.

Another very successful model organism in the study of lifespan has been the nematode *C. elegans* [51, 52]. Using RNAi by feeding [53] via large-scale libraries which cover most worm ORFs [54-56], several screens for increased lifespan have been completed [57-60]. As in *S. cerevisiae*, ribosomal components and genes involved in translation were well represented [42]. Mitochondrial genes [57, 58], largely those involved in oxidative phosphorylation, were also identified (see the accompanying review by Hwang *et al*. in this issue [61]). In cases where these screens cover overlapping worm chromosomes, the concordance of their findings is lower than might be expected. These differences are definitely due in part to variable and imperfect efficiency of worm gene knockdown by feeding RNAi, and also differences in the protocols used in each screen (time of onset of RNAi treatment being a prime example). The possible reasons for these differences have been discussed in more detail previously [62, 63]. Following the initial screens, additional work was done to look at genes whose RNAi would not allow worms to successfully reach adulthood, excluding them from the earlier findings [64, 65]. In these two studies, both groups once again found components of the ribosome and genes involved in the regulation of translation to be overrepresented, among others. Although there is some striking overlap, what is clear from the lack of more complete concordance in positive hits is that these screens have not yet saturated discovery of most or all lifespan affecting hypomorphs in *C. elegans*. This provides a strong motivation to leverage other methods to complete a saturating screen for this phenotype. As in the case of *S. cerevisiae*, this may now be possible to do in a very direct way by using recently developed high-resolution, high-throughput methods described in the next section. In the meantime it will also be important to leverage data from other species, including the recently completed yeast RLS screen, to suggest candidates for higher resolution testing for worm lifespan when orthologs can be identified. Interestingly, in comparing the two species described thus far, deletions shown to extend the lifespan of *S. cerevisiae* are significantly more likely to also extend the lifespan of *C. elegans*, quantitatively confirming a high degree of conservation of these genes and pathways [66, 67]. A whole-genome screen has also been done to identify genes necessary for the extended lifespan of *daf-2* mutants using similar methods [68].

The screens mentioned so far have involved deletions or hypomorphs. In *Drosophila*, a Gene Search [69] gain-of-function screen identified *wdb*, the PP2A regulatory subunit, as well as the serine-threonine kinase *lkb1* [70]. In addition, a misexpression screen [71] identified 45 genes including many involved in translation, as well as some involved in transcriptional regulation such as the *gug* histone deacetylase, and one surrogate screen for increased resistance to multiple stresses [72] identified a lifespan-extending effect from reduced expression of *mas1* alpha 1,2 mannosidase 1, with another identifying heat shock proteins again, among others [73]. An early screen for *Drosophila* lifespan using P-element insertion [74] identified the methuselah (*mth*) gene.

## WHOLE GENOME EXPRESSION PROFILING OF AGING AND LIFESPAN

Another approach that has been used to look at aging at the genome level is whole genome expression profiling [75] (see also the accompanying review in this issue from Hou *et al. *[76]). Two broad classes of these studies are apparent. The first is studies that look at transcriptome-wide changes as organisms age. These results naturally suggest useful biomarkers, as well as the underlying changes (which cannot always be assigned to cause vs. effect) present during natural aging. Recently, Budovskaya *et al.* used microarrays to identify 1294 genes that change during aging in *C. elegans*, and found that many of them were regulated by a shared *elt-3*/*elt-5*/*elt-6* transcriptional circuit [77], while Viñuela *et al.* have shown 1772 genes with increased variation during aging in *C. elegans* [78] using eQTL. Previous work studying whole-transcriptome changes has shown a decrease in heat-shock protein expression and an increase in transposase expression over the course of worm aging [79]. Within a genetically identical population of animals raised in a controlled homogeneous environment, there is a distribution of lifespans among individuals, and the source of the variation is not fully understood. Golden *et al.* have performed microarray analysis of individual wild type and *daf-2* hypomorph worms during aging, finding among other things that again heat-shock proteins show significant variation with age [80, 81]. In agreement with these findings, heat shock protein *hsp-16.2* expression has been shown to predict individual lifespan in a genetically homogenous population of worms [82, 83].

Just as screens for extended lifespan have been shown to have a significant overlap between *S. cerevisiae* and *C. elegans* [66, 67], whole-transcriptome changes during aging in *C. elegans* and *D. melanogaster* have been shown to be highly correlated [84], again suggesting that findings of these genome-scale experiments may be well conserved between other distantly related organisms including humans. 

The second set of studies has looked at changes between long-lived mutants and either the wild-type organism or an appropriate short-lived control. In *S. pombe*, the overexpression of *ecl1* family genes extends chronological lifespan, and microarray analysis of these strains shows an upregulation of genes involved in sexual development and stress response [85]. In *S. cerevisiae*, loss of Isc1p shortens chronological lifespan, and microarray analysis showed an upregulation of the yeast iron regulon, possibly leading to increased hydroxyl radical levels via the iron-catalyzed Fenton reaction [86].

In *Drosophila*, whole-transcriptome analysis has been done for long-lived *Lnk* mutants [87], showing changes in IIS and Ras/MapK signalling, as well as overall lipid and carbohydrate metabolism. In mammals, whole-transcriptome data has been collected for caloric restriction [88-90] and resveratrol treatment [91], both of which have life-extending effects at least under some conditions. With caloric restriction in rats [88], upregulation of Nr4a nuclear receptors was seen, while in mice, one study saw an upregulation of genes involved in inhibition of oxidative stress, inflammation, and tumorigenesis [90], while another saw feminization of the gene expression profile in male mice [89]. With resveratrol treatment, microarray results showed a downreglation of Ras and ubiquitin pathways, as evidenced by lowered expression of Ras-GRF1, RAC3, and UBE2D3. In *C. elegans*, whole-transcriptome analysis has been done for diallyl trisulfide treatment [92], resveratrol treatment [93], long telomeres [94], and long vs. short lived *daf-12* alleles [95]. Whole genome O-Glc-NAc levels have been assayed by ChIP-CHIP in lifespan-altering *oga-1* and *ogt-1* O-Glc-NAc processing mutants [96]. Additionally, whole-genome translational profiling has been done of *ifh-1(RNAi)* worms; *ifh-1* is the *C. elegans* eIF4G [97]. The transcription factor that has been examined most closely may be DAF-16 [98], whose likely targets have been studied at the whole genome level via differential display PCR [99], SAGE [100], microarrays [101-104], ChIP [105], and DNA adenine methyltransferase identification (DamID) [106, 107].

As with the whole-genome longevity screens, there is a striking overlap between several of these studies in some specifics, and they have proved extraordinarily fruitful overall in identifying genes for further study. It is also true that there are in some cases statistically significant overlaps between, e.g., the lists of likely DAF-16 targets generated in different studies. Nevertheless, the discordance of these lists of putative targets bears some thought. As has been noted, just as in the case of the whole genome longevity screens, there are significant differences in the exact protocols used to generate biological samples in the various studies. Also significant are the large differences that these high-throughput, large data set methods have between platforms, as well as lab and equipment settings. These issues have also been commented on extensively (see e.g. [108, 109]). As with the longevity screens, this incomplete concordance suggests at a minimum that additional targets or downstream effectors probably remain to be identified. Given the inherent signal-to-noise levels of simultaneous whole genome measurements, very large numbers of replicate measurements may drive convergence, and this should be increasingly feasible as the cost and time requirements per sample continue to drop rapidly. This inter-platform and inter-lab variance that has been observed, combined with the time and cost efficiency increases, may also suggest that when attempting to combine disparate sources of longevity to identify a shared whole transcriptome or whole proteome signature, generating all data in a single lab using a consistent platform could have an edge over meta-analyzing data from a wide variety of sources.

## OTHER SYSTEMS-LEVEL INVESTIGATIONS

Increasingly, the use of other genome-scale datasets has been leveraged to optimize candidate selection for lifespan testing. With current high quality tools for mining of published data sets such as WormMart [110] and YeastMine [111], and for generation of networks based on known gene interactions such as GeneMania [112] and Cytoscape [113], as well as for identifying cross-species orthology relationships [114], network-based thinking has been increasingly applied to the study of aging and lifespan [115-118]. Recently, the novel computational method of network identification by regression (NIR) [119] has been used to identify new lifespan effects, by using transcriptional perturbations to build a model of functional interactions [118]. Although we have focused here on the most widely studied model organisms, others such as the previously mentioned *S. pombe*, *P. anserina*, and *N. crassa*, as well as the rapidly developing vertebrate model system *N. furzeri* [120, 121], will greatly contribute to this cross-species leverage in systems-level investigations of aging.

Other recent work has used metabolomics to suggest likely long-lived deletions in *S. cerevisiae *[122], by using the metabolic fingerprints of known long-lived deletion strains to generate a classifier based on an orthogonal projection to latent structure [123], which was then used to successfully predict novel long-lived deletion strains based on their metabolomics profiles.

Finally, two studies have looked at changes in protein solubility over the course of natural aging using complete-proteome surveys by iTRAQ [124, 125]. In one of these studies [125], several hundred proteins were found to become insoluble with age. These were shown to be enriched for beta sheets, and overall protein aggregation and insolubility was delayed in long-lived insulin signalling mutants. In the second study [124], 203 proteins were identified that became SDS insoluble with age, and a higher proportion (41%) of these genes’ knockdown by RNAi could extend lifespan than the proportion (18%) of a control group of genes.

## FUTURE DIRECTIONS: HIGHER RESOLUTION DATA VIA HIGHER THROUGHPUT ASSAYS

One inescapable conclusion of the aggregate results of genome-wide studies of aging to date (see summary Table **1**) is that we have not come close to saturating the number of potentially lifespan-altering genes in any organism. This is in no small part because directly generating survival curves is a relatively time-consuming process in most model organisms using current methods. There are several possible ways to address this. One way that has been tried is by attempting to find surrogate phenotypes [72, 73, 126] that can be screened more rapidly, or even scored under selection. Another is mining candidates from the many whole-genome expression profiles. Results to date with these have been very fruitful, but have not suggested that these methods alone will rapidly saturate our search for lifespan- and healthspan-altering genes in tractable model organisms.

For multiple measurements of the same genome-wide assay, repeated experiments within one lab and platform may offer an advantage to meta-analysis due to reduced variance, in particular as the cost per experiment drops rapidly. On the other hand, there is another way in which data may be leveraged synergistically: across disparate measurements. Easily queryable databases of protein-protein binding, synthetic sick / lethal interactions, and myriad other phenotypes are appearing online at an increasing rate, and increasingly becoming integrated in their data export formats. Therefore, it is increasingly possible to compare longevity or expression datasets to a wide range of other datasets, which may help cobble together the aging data into a more coherent level of understanding.

Another way forward would be to generate robust, high resolution survival curve data genome-wide for these organisms. Because of the huge number of individual lifespan experiments involved, the idea of a future where almost every gene on Wormbase, Flybase, or SGD displays a high resolution, statistically meaningful survival curve alongside its corresponding control has been until recently difficult to imagine. However, rapidly developing methods for automation of lifespan assays may soon make this level of data commonplace.

In yeast, the chronological lifespan assay has already been well automated [26, 31], and two automated, microfluidics-based methods have recently been published [127, 128] which may soon greatly increase the throughput for the yeast replicative lifespan (RLS) assay. Additionally, a genetic selection for mother vs. daughter cells has also been used to increase throughput relative to standard micro-dissection for RLS [129]. In worms, a microfluidics-based approach has recently been described [130, 131], as well as a 96-well [132] and 384-well [133] plate-based assay. When attempting to extrapolate gains in high throughput data, it is important not to underestimate the exponential increase in capability provided by rapid advances in the underlying nascent technologies; remember that in the case of the human genome, an optimistic early estimate for completion was 2050. The rate of increase in technological advancement provides a great deal of optimism that intense focus on aging will yield a systems level understanding of this complex process in a not-too-distant timeframe.

## Figures and Tables

**Table 1.** Genome-Scale Studies of Aging

Authors	Year	PMID	Organism	Summary
Powers, R.W. 3rd *et al.*	2006	16418483	*S. cerevisiae*	Measurement of chronological lifespan in approximately 4800 yeast single-gene deletion strains.
Matecic, M. *et al.*	2010	20421943	*S. cerevisiae*	Microarray-based pooled screen for chronological lifespan.
Kaeberlein, M. *et al.*	2005	16293764	*S. cerevisiae*	Screen of a subset of the yeast deletion collection for altered replicative lifespan.
Dillin, A. *et al.*	2002	12471266	*C. elegans*	Lifespan screen of Chromosome I using the Ahringer RNAi library.
Lee, S.S. *et al.*	2003	12447374	*C. elegans*	Lifespan screen of Chromosomes I and II using the Ahringer RNAi library.
Hamilton, B. *et al.*	2005	15998808	*C. elegans*	Lifespan screen of 16,475 RNAi clones using the Ahringer RNAi library.
Hansen, M. *et al.*	2005	16103914	*C. elegans*	Lifespan screen of 13,417 RNAi clones using the Ahringer RNAi library.
Curran, S.P., *et al.*	2007	17411345	*C. elegans*	Lifespan screen of 2700 genes essential for viability by RNAi at adulthood.
Chen, D. *et al.*	2007	17521386	*C. elegans*	Lifespan screen of 57 genes with a larval arrest RNAi phenotype by RNAi at adulthood.
Samuelson, A.V. *et al.*	2007	18006689	*C. elegans*	Genome-wide RNAi screen for suppression of the extended lifespan of daf-2 mutants.
Funakoshi, M. *et al.*	2011	21281604	*D. melanogaster*	Gain-of-function lifespan screen using the gene search system.
Paik, D. *et al.*	2012	22366109	*D. melanogaster*	Lifespan screen by EP-element misexpression.
Budovskaya, Y.V. *et al.*	2008	18662544	*C. elegans*	Identification of 1294 genes whose expression changes with age from whole-transcriptome array data.
Viñuela, A. *et al.*	2010	20488933	*C. elegans*	Identification of 1772 genes with increased variation with age using eQTL.
Lund, J. *et al.*	2002	12372248	*C. elegans*	Whole-transcriptome examination of expression changes during aging.
Yoshida, R. *et al.*	2010	20550517	*S. cerevisiae*	Classification of likely long-lived yeast deletions by metabolomic fingerprint.
Reis-Rodrigues, P. *et al.*	2012	22103665	*C. elegans*	Global analysis of protein solubility during aging using iTRAQ
David, D.C. *et al.*	2010	20711477	*C. elegans*	Global analysis of protein aggregation during aging using iTRAQ
